# Risk factors leading to preterm births in Morocco: a prospective study at the maternity Souissi in Rabat

**DOI:** 10.11604/pamj.2015.22.21.5100

**Published:** 2015-09-10

**Authors:** Nargisse Sabiri, Meryem Kabiri, Rachid Razine, Amina Barkat

**Affiliations:** 1 Service medicine and neonatal resuscitation, Children's Hospital, University Hospital, Rabat, Morocco; 2 Research Team on Health and Nutrition of Mother and Child, University Mohammed V Souissi, Rabat, Morocco; 3 Research Center in Clinical Study and Therapeutic Rest, Faculty of Medicine and Pharmacy, Rabat, Morocco; 4 Laboratory for Biostatistics and Clinical and Epidemiological Research, Faculty of Medicine and Pharmacy, Rabat, Morocco

**Keywords:** Preterm infants, risk factors, mortality

## Abstract

**Introduction:**

Eminent morbidity and mortality of preterm infants is perceived, especially in developing countries. The aim of the study is to identify the main factors involved in the occurrence of premature births in Morocco.

**Methods:**

This was a descriptive and analytical study conducted at the maternity Souissi in Rabat, from January 2011 to December 2011. The data were collected using interview with women in the postpartum, and via, the exploitation of obstetric and perinatal records. The data sheet was filled out for each newborn, including socio-demographic, obstetrical, maternal, childbirth and neonatal data, as well as, monitoring and surveillance of pregnancy.

**Results:**

A total of 1015 births were collected. 954 were full term babies and 61 were preterms. The gestational age was between 33-34 weeks in 57.4%. Relying on Statistical analysis, many risk factors were, significantly, associated with the occurrence of prematurity, namely: low level of maternal education (p < 0.004), absence of pregnancy’ monitoring (p < 0.001), multiparity (p < 0.001), maternal chronic diseases (p < 0.001), and drug taking during pregnancy (p < 0.001).

**Conclusion:**

To reduce the incidence of preterm births, reliable programs must be established, devoting all its interest, to educate the young woman in childbearing age about the appropriate ways of monitoring pregnancy, as well as, the qualitative and quantitative development of health care structures.

## Introduction

Prematurity leads to eminent mortality and even higher neonatal morbidity, despite the advance in neonatology. The struggle, therefore, remains a major concern, especially in developing countries, where modern neonatal intensive care techniques are not always available. The socio-economic outcome consecutive to this phenomenon (birth, economic cost) is not insignificant. Thus; the objective of our study is to identify the major factors leading to prematurity, and to deduce some Guidelines for health and social policy in this context.

## Methods

This was a prospective descriptive and analytical study conducted at the maternity Souissi in Rabat, from January 2011 to December 2011.

### Setting

Maternity Souissi is a tertiary care unit that receives expectants from all regions of the kingdom. The annual number of births is at an average of 15,000.

### Methods

The collection of data was done in two steps. Firstful, the socio-demographic status of women, their prenatal care, and their behavior during pregnancy were obtained by interviewing women in postpartum before leaving the maternity. Secondly, the processes of childbirth and child health at birth were identified from medical records. A data sheet was completed by the physician for each newborn, including: newborns’ features: weight, sex, term of pregnancy; identity and socio-professional status of the mother: age, occupation, education, origin, place of residence; antenatal and neonatal data: parity, number and place of antenatal consultations, diseases observed during pregnancy, place and mode of delivery, birth weight, Apgar scale, number of children from the same pregnancy, congenital malformations, and drug taking during pregnancy.

### Inclusion criteria

All births occurring from 9am to 12am on working days, whatever the mode of delivery or the term of pregnancy were recruited. This timing was settled as a result of lacking in the number of physicians necessary to fill in all the data sheets, which include more than 160 items.

### Definition

Prematurity is defined as any birth before 37 weeks of gestation (WG), or before 259 days, counting from the first day of last menstrual period: premature is defined as any baby born between 32 and 36 weeks of gestation; very premature defines any baby born before 32 weeks of gestation; very low birth weight corresponds to any baby born before 28 weeks of gestation; pregnancy is monitored, if there were at least three antenatal consultations. A woman is said multiparous, if she has 4 children or more, and paucipare if she has 2 or 3 children. The adaptation to extra uterine life is good, if the Apgar scale is greater than or equal to 7 in the first 5 min of life. The educational level of women was classified into the following classes: illiterate, primary, secondary and higher educational level.

### Data analysis

A correlative investigation of the maternal characteristics, using a logistic regression model, was conducted to determine the specific role of each in the maternal risk of prematurity. All the data were entered in SPSS version 13.0 for analysis. Quantitative variables were expressed as mean and standard deviation. Categorical variables were expressed in numbers and percentages. Quantitative variables were compared using Student′s test and the qualitative variables by Chi 2 or Fisher′s exact test. The level of statistical significance was chosen to p <0.05.

## Results

### Incidence

On a total of 1015 deliveries, 61 preterm births were registered, giving an incidence of 60 per 1000 live births: very premature meaning all births before 32 WG, and representing 1.67% of all births; premature represented 3.44% of all births, and that occurring between 35 and 37 weeks of gestation represented 0.88%.

### Maternal data (Table 1)

Socio-demographic features: among the group of preterm infants, 311% were housewives women without constant income, and 11.4% were in the group of full term newborns. The rate of women from rural areas was 23% in the group of preterm babies and 8.7% in the group of full term newborns.

**Table 1:** Maternal, obstetrical and socioprofessional features

	Preterm newborns N = 61	Full term newborns N = 954	P
**Place of residence Rural**	14(23%)	83(8,7%)	0,003
Urban	43(70,5%)	799(83,8%)	
Periphery	4(6,5%)	72(7,5%)	
Mother's origin Arab	52(85,2%)	778(81,6%)	0,21
Berber	9(14,8%)	176(18,4%)	
**Age**			<0,001
≤20 years	22(36,1%)	83(8,7%)	
21-35 years	30(49,2%)	756(79,2%)	
>35 years	9(14,8%)	115(12%)	
**Level of education**			<0,001
Illiterate	38(62,3%)	304(31,9%)	
Primary	13(21,3%)	283(29,7%)	
Secondary	8(13,1%)	309(32,4%)	
Universitary	2(3,3%)	58(6,1%)	
**Maternal activity during pregnancy**	19(31,1%)	109(11,4%)	<0,001
**Parity**			0,002
primiparous	42(68,9)	451(47,3%)	
Paucipare	12(19,7%)	415(43,5%)	
multiparous	7(11,5%)	88(9,2%)	
Drug taking during pregnancy	18(29,5%)	84(8,8%)	<0,001
**Pregnancy’ monitoring**			<0,001
Yes	27(44,3%)	838(87,8%)	
No	34(55,7%)	116(12,2%)	
Maternal chronic diseases	31(50,8%)	102(10,7%)	<0,001
**Mode of delivery**			0,25
Ceasarean	12(19,7%)	131(13,7%)	
Vaginal delivery	49(80,3%)	823(86,3%)	
**Number of babies in the same pregnancy**			0,002
Unique	49(80,3%)	885(92,8%)	
Multiple	12(19,7%)	69(7,2%)	

Age: the mean age was 27 ± 7 years in the group of preterm infants and 27.7 ± 6 within the group of full term newborns. The majority of women were aged between 21 and 35 years (49.2% within the group of preterm babies and 79.2% within the group of full term newborns). In 36.1% of cases of prematurity, the age was less than or equal to 20 years, and above 35 years in 14.8%.

Education: 62.3% of women were illiterate within the group of preterm infants, and 31.9% were within the group of full term newborns.

Parity: primiparous women were more represented in the group of preterm infants with a proportion of 68.9%, followed by paucipares (19.7%) and multiparous (11.5%). While in the group of full term babies the rates were respectively 47.3%, 43.5% and 9.2%.

Medications or herbs: in the group of preterm infants, The rate of women who took drugs (antiepileptic) and / or plants (Fenu Greek), during pregnancy was 29.5%,whereas, 8.8% were within the group of full term babies.

Mode of delivery and pregnancy features: twin pregnancy was in 19.7% of cases within the group of preemies and 7.2% within the group of full term newborns. The rate of vaginal delivery was, almost, the same in both groups (80.3% within the group of preemies and 86.3% in full-term babies).

Pregnancy monitoring: the pregnancy was monitored in 44.3% within the group of preemies. Also, 50.8% of women suffered from chronic diseases, namely, high blood pressure (hypertension). While, in the group of full-term newborns, 87.8% of pregnancies were monitored. And only, 10.7% of women suffered from chronic diseases (hypertension).

### Neonatal data (Table 2)

Adaptation to extra-uterine life: 21.3% of preterm newborns do not have a good adaptation to extra uterine life, with an Apgar less or equal to 7, in the fifth minute of life. It was less or equal to 7, in the first minute of life, in 37.7%.

**Table 2:** Neonatal features

	Preterm newborns N = 61	Full term newborns N = 954	*P*
**Apgar 1min**			<0,001
^3^7	38(82,3%)	921(96,5%)	
<7	23(37,7%)	33(3,5%)	
**Apgar 5min**			<0,001
^3^7	48(78,7%)	940(98,5%)	
<7	13(21,3%)	14(1,5%)	
**Sex**			0,35
M	28(45,9)	504(52,8%)	
F	33(54,1%)	450(47,2%)	
**Weight**			<0,001
<1500g	32(52,5%)		
1500-2000g	24(39,3%)		
>2000g	5(8,2%)	954(100%)	
Congenital anomalies	8(13,1%)	32(3,4%)	0,002
Deaths in first 24h	8(13,1%)	23(2,4%)	<0,001

Weight: the average weight was 1504 ± 372. The majority of preemies (91.8%) had a birth weight of 2000g or less, and 52.5% were less than 1500 g.

Evolution and prognosis: the preemies weighing over 2000 g required no special care in 8.2% of cases, and they were given back to their mothers from the first day. The others were hospitalized in the neonatal unit. During the study, eight deaths were recorded, implying a mortality rate of 13.1%. This mortality was, especially, noted in the early neonatal life. The malformation rate was 13.1% for preemies and 2.4% for full term newborns.

### Risk factors associated with prematurity (Table 3)

An accurate analysis of all factors studied leads us to retain only the risk factors, significantly, associated with prematurity, essentially: maternal age greater than 35 years (p = 0.004), multiparity (p = 0.039), drugs taking during pregnancy (antiepileptic) and / or plants (Fenu Greek) (p < 0.001), maternal chronic diseases (HTA) (p< 0.001), rate of malformations (p < 0.001), twins (p < 0.001), the mothers’ activity during pregnancy (p <0.001) and non-monitoring of pregnancies (p < 0.001). However, young women (under 20 years), with low level of education or from rural areas, Arab or Berber were not considered as risk groups.

**Table 3:** Risk factors associated with prematurity: multivaried analysis

	Odd Ratio	Confidence interval CI 95%	*P*
Maternal origin	2	0,70-5,66	0,19
Place of residence	0,69	0,46-1,04	0,07
**Age**			
≤20years	1,98	0,92-4,29	0,08
>35years	0,29	0,13-0,68	0,004
Maternal activity during pregnancy	3,28	1,84-5,85	<0,001
Parity primiparous	0,85	0,37-1,96	0,71
Multiparous	2,75	1,05-7,18	0,039
drug and/or plants taking during pregnancy	4,33	2,39-7,85	<0,001
Pregnancy'monitoring	0,11	0,064-0,189	<0,001
Maternal chronic diseases (hypertension)	8,63	5,01-14,84	<0,001
Multiple pregnancies	3,14	1,59-6,18	0,001
Congenital malformations	4,34	1,91-9,90	<0,001
Low level of education	0,27	0,06-1,17	0,08

## Discussion

The present study demonstrates that the decrease in the incidence of prematurity is associated with providing appropriate strategies against the identified risk factors. In literature, the average frequency of preterm births is estimated at 66 per 1000, with extremes ranging from 16.7 to 247 for 1000 [1]. This frequency was 60 per 1000 in our study; and, it remained stable for several years between 6-7%. In most works, factors such as twins and high blood pressure were traditionally found [1–4]. It is the same for grand multiparity, history of preterm delivery or curetting for abortion [5]. Also, Environmental factors, such as overloading domestic labor and drug or plants taking (antiepileptic and/or Fenu Greek, an oldest medicinal and culinary plant) have, certainly, a significant role in increasing prematurity in our developing country. To confirm these data, further researches are needed.

In the Nineties, the proportion of mothers aged below 20 years continued to decline. These young mothers were no more at high risk in comparison to women over 35 years [6]. The same thing has been reported in several countries, such as Finland [7] and Sweden [8], where the births rate of women below the age of 20 is very low. In the present study, the age over 35 years is a risk factor for preterm births. This fact could be explained by maternal chronic diseases, in late pregnancies, namely hypertension. Hence, preterm fetal extractions were made in the interest of the child and the mother.

The WHO recommends, at least, four antenatal consultations (ANC), during pregnancy [9]. The lack of ANC, found in 55.7% of cases, was confined to developing countries. The low number of ANC in our regions increases the risk of having a preterm infant [10, 11]. In fact, it is during ANC that mothers receive counseling related to pregnancy and childbirth, and potential risks of pregnancy can be prevented, detected in time and caught early.

### Prevention

Medical policy: primary prevention is expected to act on crucial causes via early medico-social awareness of risk pregnancies [12, 13]. This prevention will, also, influence the environment, through the training of health professionals in charge of prenatal care, as well as, the information of young women on pregnancy risk factors.

Knowing the etiological factors allowed some authors to create different scales, in order to predict preterm births [14, 15]. These scales, essentially preventive, allowed the screening of all pregnancies at high risks. The best known and most convenient to use seems to be the coefficient of premature delivery risk (CPDR) by Papiernik [14]. A screening policy based on CPDR scales, during the first prenatal consultation (especially about twin pregnancy and hypertension) would improve the staff awareness about the issue, and promote the prevention against prematurity.

Social policy: in our context, where women endure difficult conditions of life and suffer from high rate of illiteracy, preventive measures should be established, targeting: women empowerment, improving access to education, economic relief, workload alleviation (especially in rural areas). Besides, national concerns should shed light to limit the consumption of Fenu Greek plants.

## Conclusion

Preterm birth remains a major cause of premature morbidity and mortality. The high incidence of mortality and high economic costs are significant damages that burden the health care system. Hence, reliable and accurate strategies must be established, through preventive programs, in order to reduce the incidence of adverse outcomes. These preventive programs would involve the development of a social protection of the pregnant women, as well as, the qualitative and quantitative development of healthcare structures; in addition to, education, information, and communication with young women about the proper way of pregnancy monitoring.
